# Oleoylethanolamide: A Novel Potential Pharmacological Alternative to Cannabinoid Antagonists for the Control of Appetite

**DOI:** 10.1155/2014/203425

**Published:** 2014-04-03

**Authors:** Adele Romano, Roberto Coccurello, Giacomo Giacovazzo, Gaurav Bedse, Anna Moles, Silvana Gaetani

**Affiliations:** ^1^Department of Physiology and Pharmacology “V. Erspamer”, Sapienza University of Rome, Piazzale Aldo Moro 5, 00185 Roma, Italy; ^2^Institute of Cell Biology and Neurobiology (IBCN), Italian National Research Council (CNR), Via del Fosso di Fiorano, 64-00146 Roma, Italy; ^3^Genomnia Srl, Via Nerviano 31, Lainate, 20020 Milano, Italy

## Abstract

The initial pharmaceutical interest for the endocannabinoid system as a target for antiobesity therapies has been restricted by the severe adverse effects of the CB1 antagonist rimonabant. This study points at oleoylethanolamide (OEA), a monounsaturated analogue, and functional antagonist of anandamide, as a potential and safer antiobesity alternative to CB1 antagonism. Mice treated with equal doses (5 or 10 mg/kg, i.p.) of OEA or rimonabant were analyzed for the progressive expression of spontaneous behaviors (eating, grooming, rearing, locomotion, and resting) occurring during the development of satiety, according to the paradigm called behavioral satiety sequence (BSS). Both drugs reduced food (wet mash) intake to a similar extent. OEA treatment decreased eating activity within the first 30 min and caused a temporary increase of resting time that was not accompanied by any decline of horizontal, vertical and total motor activity. Besides decreasing eating activity, rimonabant caused a marked increase of the time spent grooming and decreased horizontal motor activity, alterations that might be indicative of aversive nonmotivational effects on feeding. These results support the idea that OEA suppresses appetite by stimulating satiety and that its profile of action might be predictive of safer effects in humans as a novel antiobesity treatment.

## 1. Introduction

Despite the continuous increase of obesity incidence in all developed countries, limited pharmacological therapies are currently available to treat obesity in an efficacious and safe manner. Thus, development of effective and safe anti-obesity therapies is a priority for both patients and health systems. Efficacy and safety are the ideal endpoints of anti-obesity medications that should put together the ability to suppress food intake and reduce fat depots with the increase of nutrients oxidation and lack of major side effects burden. One of the most promising novel pharmacological targets for drug development is the endocannabinoid (EC) system and its related compounds, the N-acylethanolamines (NAEs). Among the latter ones, anandamide represents the most studied compound mainly for its ability to act as endogenous ligand for cannabinoid receptor type 1 (CB1). A large body of evidence has clearly demonstrated that the stimulation of this receptor exerts powerful effects on energy balance, increasing appetitive drive and promoting adiposity [1, 2]. Therefore, several CB1 receptor antagonists have been tested in both genetic and dietary rodent models of obesity for their potential effects as antiobesity treatments [3–9]. One of these compounds, rimonabant (also referred as SR141716A) reached the market after successful clinical trials, which confirmed the observations made in rodents on its efficacy in body weight reduction and in the metabolic benefits induced in obese subjects [10, 11]. However, the emergence of severe psychiatric adverse effects, mostly attributable to the inverse agonistic activity of rimonabant at central CB1 receptor, led to its withdrawal from the market [12, 13]. Such drawbacks led to a reassessment of the potential of the EC system as a drug target for novel antiobesity therapy aimed at preventing the severe side effects induced by central CB1 receptors blockade. Several reports have been indicating as possible safer pharmacological alternatives to rimonabant a number of novel “neutral” or “silent” CB1 receptor antagonists, such as AM4113, peripherally restricted CB1 receptor antagonists, such as AM6545, LH-21, MJ15, URB447 [14], CB1 receptor partial agonists, allosteric modulators of CB1 receptors, and other agents able to alter EC levels [15].

In this context, increasing interest has been focusing also on a group of NAEs that are AEA congeners but seem to act through mechanisms independent of CB1 receptors. This group includes the monounsaturated analogue oleoylethanolamide (OEA) [16, 17], which, although sharing similar biosynthetic pathways [18] with AEA, exerts opposite effects on feeding regulation and lipid metabolism. AEA binds with high affinity the CB1 [19] and its administration induce hyperphagia that can be attenuated or abolished by CB1 receptor blockade [20–23]. In contrast to AEA, OEA does not have any affinity for CB1 receptor and its systemic administration inhibits food consumption in rodents by delaying eating onset. Recent studies on the physiological role of OEA demonstrated that it may act as a gut-derived satiety factor (for review: [16, 17, 24, 25]). In particular, OEA is synthesized in the upper part of the small intestine, upon the absorption of lipids from the diet [26–28]. OEA binds with high affinity the nuclear receptor peroxisome proliferator-activated receptor *α* (PPAR-*α*) [29] to control the expression of several genes involved in fat absorption and fatty acid metabolism, to activate hypothalamic oxytocinergic neurons and to inhibit further eating [29–32]. These effects were observed in rodents when OEA was systemically administered at dosages ranging from 5 to 20 mg/kg [32, 33], with an ED50 = 9.2 ± 1.6 mg/kg, i.p. [34].

Accumulating evidence suggests that OEA might be involved in different pathophysiological aspects of appetite and metabolism regulation. For example, elevated OEA concentration has been described in plasma and cerebrospinal fluid of woman affected by eating disorders [35], in the saliva of obese subjects [36], and in the subcutaneous adipose tissue of subjects with both obesity and type 2 diabetes [37]. Conversely, decreased OEA intestinal content was observed in rodent models of obesity [38–40]. These findings might suggest that excessive or altered food intake may render the mechanism dysfunctional, raising the possibility that OEA might represent a novel pharmacological target to treat such pathologies. This hypothesis is supported by several observations made on obese rodent models in which OEA decreased hyperphagia and body weight gain, increased lipolysis, and decreased hypertriglyceridemia, hypercholesterolemia, and liver steatosis, when chronically administered thus demonstrating a significant effect not only on the acute, short-term—but also on the long-term appetite and energy regulation [31, 41–45]. Moreover, the link between dietary fat intake and OEA's action on feeding behaviour and obesity has been recently strengthened by the finding that OEA subchronic treatment can reestablish a normal response of the brain reward system to an intraduodenal infusion of lipids that seems to be altered in diet-induced-obese mice [46]. Since similar reward hypofunctionality has been described in obese subjects and has been proposed as part of the central mechanism sustaining hyperphagia, this finding suggests a potential therapeutic relevance of OEA in food addiction [46]. Based on these considerations, in this study we propose OEA as a possible pharmacological alternative to anorexiant and/or antiobesity drugs targeting CB1 receptors. To test our hypothesis we compared the acute effects of OEA with those of rimonabant on spontaneous behaviors observed in mice during the beginning of the consummatory phase of their daily activities, as assessed in the paradigm of the behavioral satiety sequence (BSS).

The commonest dependent variable recorded in animal studies aimed at understanding drug effects on feeding is the amount of food eaten by the animals [47]. However, food intake and feeding behavior can be affected by several factors, including those acting in nonspecific manners, such as pain, stress, anxiety, and nausea. Therefore, one of the most contentious issues in the psychopharmacology of appetite regulation is the identification of the physiologic mechanisms underlying the observed reduction of eating. Animals cannot report their aversive side effects and the maintenance of a normal feeding structure in strings of behavioral acts may be used to verify drug effects on the normal physiology of appetite regulation. The BSS is an observational approach that allows investigating the effects of drugs acting on feeding by examining the presence of an orderly progression of behavioral patterns from eating to grooming and finally to resting, as it is typically observed in the rodent spontaneous behavior during the development of satiety [48, 49]. As previously underlined [50, 51], since the BSS relies on the animal's spontaneous pattern of feeding, this paradigm helps to circumvent several problems of model validity associated to other testing procedures.

In previous studies, the analysis of BSS in rats treated with rimonabant has revealed that this compound preserves the order of events and, nevertheless, differs markedly from the natural satiation. The most notable difference is that grooming (particularly scratching) is profoundly enhanced at anorectic doses, while eating and resting are diminished, raising the possibility that anorectic effect is simply secondary to the grooming effect [50, 52]. Recent evidence does not appear to support this hypothesis, showing that the acute anorectic response induced by rimonabant cannot be just accounted by the time spent in grooming behavior [14, 53]. Moreover, excessive grooming in rodents may be indicative of an altered emotional state induced by rimonabant administration. This hypothesis has been confirmed by specific behavioral tests in rodents and by clinical observations showing that rimonabant is an anxiogenic compound [54, 55].

On the other hand, several clues suggest that the anorexiant actions of OEA are not attributable to stress or malaise. In fact, it has been shown that OEA does not elicit fear or anxiety-like behaviors, does not affect plasma corticosterone levels, and does not induce conditioned taste aversion in rats [31]. Moreover, by the meal pattern analysis of animals subjected to the acute OEA treatment, we previously demonstrated that OEA selectively delays the normal eating onset in free-feeding rats and mice without affecting meal size and postmeal interval, suggesting that this lipid mediator may participate in the physiological control of satiety [32]. However, whether OEA can affect other spontaneous behaviors related to eating, such as those analyzed in the BSS paradigm, remained unexplored [33].

Therefore, in this study we evaluated the behavioral profile of mice that underwent acute administration with either OEA or rimonabant, by focusing on the occurrence of spontaneous behaviors typically investigated via the BSS paradigm. Per each compound we tested two different doses (i.e., 5 or 10 mg/kg, i.p.), based on the results of our previous acute dose-response studies [32–34] on OEA's anorexiant effects. Both OEA doses were previously demonstrated to be sufficient to activate fully PPAR-*α* [29]. The dosages of rimonabant were chosen to obtain an acute inhibition of food intake in mice that could be similar to that elicited by OEA treatment. These doses are consistent with previous studies [4, 56–59] showing that they can lead to more than 95% of brain CB1 receptor occupancy [60].

## 2. Materials and Methods

### 2.1. Animals and Housing

All experiments were carried out on male C57BL6/J inbred mice that were generously provided by the CNR-EMMA animal facility (Monterotondo, Rome, Italy). Mice, 9-week-old, weighing 26 ± 0.5 g at the beginning of the experiments, were housed individually in breeding cages (26.7 × 20.7 × 14 cm) in a room with humidity control and constant temperature (22 ± 1°C) on a 12 h light/dark cycle (lights off 7:00 PM). Animals had ad libitum access to standard diet (4RF21; Mucedola s.r.l., Milan, Italy) and water. Thirty-eight C57BL6/J mice were used for the study of feeding behavior via the BSS protocol. Housing, animal maintenance, and all experiments were performed in accordance with the Council Directive of the European Community (86/EEC) of the Italian D.L. 116 (January 27, 1992) and approved by veterinarian supervision.

### 2.2. Drugs

*N*-Piperidino-5-(4-chlorophenyl)-1-(2,4-dichlorophenyl)-4-methyl-pyrazolecarboxamide (rimonabant) and (9Z)-*N*-(2-hydroxyethyl)octadec-9-enamide (OEA) were dissolved in 5% Tween—80/5% polyethylene glycol/saline (by volume) and injected intraperitoneally (i.p.) in a volume of 10 mL/kg of body weight. Rimonabant and OEA were purchased from Cayman Chemical (Ann Arbor, Michigan 48108, USA). All HPLC chemicals were from Sigma-Aldrich (Milan, Italy).

### 2.3. Habituation to Wet Mash

Before the start of BSS, mice were habituated for 10 days to the special diet used during the behavioral satiety sequence procedure (see below), which consists in a nonsweetened wet meal (i.e., wet mash, WM) that was always prepared fresh on a daily basis. In particular, wet mash was made up of a mixture of one-part ground standard dry powdered food pellets to 2.5 parts distilled water, during both habituation and BSS testing. Powdered food was obtained by the standard maintenance diet (4RF21 diet) that provides an energy value of 3.95 Kcal/g. The use of hydrated food or WM as test diet has been acknowledged in the BSS by several studies and procedures [61–63]. WM provides higher than standard diet palatability also in its nonsweetened form. The increase in palatability provides higher mean diet intake as compared to nonhydrated food and its consistency permits to minimize spillage, thus allowing a more accurate estimation of food intake. Although spillage is maximally curtailed by WM texture, its possible occurrence was always checked at the end of each observational period. WM was provided in small opaque plastic beakers (3 cm diameter) mounted on a plastic Petri dish [48], further reducing the possibility that WM is consumed in a different place. During the habituation phase, WM was offered for 2 h/day during daylight period (4:00–6:00 PM) up to the day before drug treatment and BSS testing. During the habituation to WM consumption, both body weight (g) and food intake (g) were measured daily (data not shown). The intake of wet mash was the difference between the weight of WM-containing food dispenser immediately before the meal presentation and the remaining food collected and recorded after 2 h. The BSS analysis was performed only in case WM daily consumption stabilized over the course of the habituation period, thus preventing the risk of neophobic reactions.

### 2.4. Drug Treatment and Behavioral Satiety Sequence (BSS)

According to a former analytic description of the BSS procedure [51], all the behavioral patterns were monitored continuously and not coded or collected by a sampling technique. The duration (sec) of the behavioral patterns (i.e., time spent in a selected behavior) was recorded for each subject and separately scored, as follows:* eating activity* (mouse leaned on food dispenser biting, chewing, or holding food in paws),* grooming* (face and body cleansing, i.e., mouse scratching its face with its forepaws, licking or biting the coat),* resting* (total immobility),* locomotion* (horizontal motor activity),* rearing* (mouse completely erected on its hind legs), and total* motor activity* (horizontal activity and rearing). BSS-associated behaviors were recorded in animals' home cages, thus avoiding the need of preliminary habituation to the testing environment to minimize novelty-induced anxiety. Trained observers blind to the experimental conditions performed the behavioral scoring. Mice were fasted for 12 h (08:00 AM–08:00 PM) and the evolution of BSS was recorded during the dark cycle (08:00 PM-09:00 PM) in a soundproof cubicle equipped with an infrared night-vision video recording camera (Panasonic color CCTV Camera WV-CP310/G). On the basis of a number of studies documenting the mean interval of satiety progression in rodents [48, 51, 64], the procedure was stopped after 45 min. This testing duration matches the time window described in the analysis of a typical BSS experimental protocol [62] that designates 40 min of feeding during the light-off phase as the time interval during which rodents ingest the most part of their daily food intake and the effects of anorectic agents can be better quantified. The testing duration of BSS is compatible with (i) the duration of OEA's anorexiant action that was previously reported to be evident for 1 h after treatment at the same doses used in the present study, (ii) the maximum plasma levels concentration of OEA (at either 5 mg/kg or 10 mg/kg) detected 30 min after its administration [32, 34, 65], and (iii) the in vivo half-life of 118.9 (±66.1) min of rimonabant, when administered at 1 mg/kg [66].

Immediately before wet mash presentation, mice were administered with one of the following treatments: OEA (either 5 or 10 mg/kg, i.p.; *N* = 8 and *N* = 8, resp.), rimonabant (either 5 or 10 mg/kg, i.p.; *N* = 8 and *N* = 8, resp.), or vehicle (10 mL/kg, i.p.; *N* = 6). Mice were given a preweighed amount of wet mash with the difference between initial and final weights corresponding to wet mash intake.

### 2.5. Data Analysis

Statistical analysis was performed using StatSoft, Inc. (2007) STATISTICA, version 8.0.

The total amount of wet mash consumed by animals during the 45 min observation period was analyzed by one-way ANOVA. The same statistical analysis was adopted to analyze the total duration of eating activity during the 45 min period. To analyze behavioral changes during the BSS, the 45 min continuous recording for each behavioral category was partitioned in 3 × 15 min time intervals. OEA and rimonabant effects on the duration of each BSS behavior were assessed by two-way repeated measures ANOVAs with treatment (5 levels, comprising two doses per drug and one vehicle group) as between-subject variable and time (3 levels, including 3 time intervals of 15 min each) as within-subject variable. All post hoc comparisons were carried out by Tukey HSD test. The criterion value for all statistical tests was *P* < 0.05.

## 3. Results

### 3.1. Effects of OEA and Rimonabant Administration on Wet Mash Intake and Time Spent Eating

**Figure 1:**
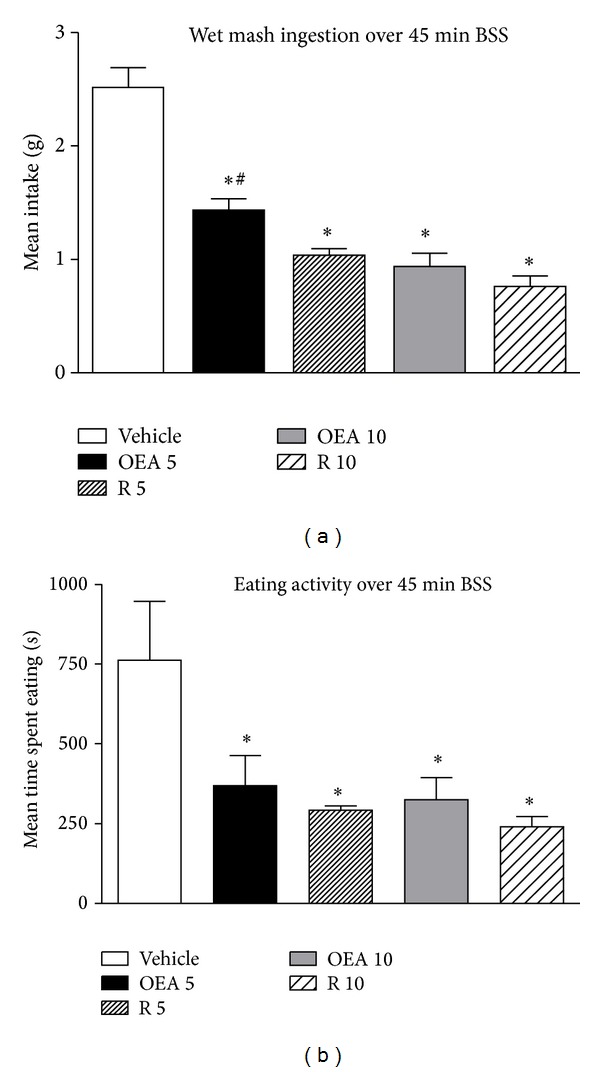
(a) and (b) show, respectively, mean cumulative wet mash intake (±S.E.M) and mean time spent in eating activity (±S.E.M) by mice treated with vehicle, OEA 5 mg/kg i.p. (OEA 5), rimonabant 5 mg/kg i.p. (R 5), OEA 10 mg/kg i.p. (OEA 10), and rimonabant 10 mg/kg, i.p. (R 10) over the 45 min BSS, (*n* = 6 vehicle group and *n* = 8 both doses of rimonabant and OEA-treated mice). *P < 0.05 versus vehicle; ^#^ *P* < 0.05 versus OEA 5 (Tukey HSD test).

The one-way ANOVA of the total amount of wet mash consumed showed a significant effect of treatment (*F*_4,33_ = 37.50, *P* < 0.0001). The post hoc analysis revealed that both drugs affected this parameter to a similar extent (Figure 1(a)). More specifically, a dose-dependent inhibition of mash intake was observed after OEA administration, while rimonabant treatment similarly inhibited feeding at both dosages (Figure 1(a)). Treatments affected also the total time spent eating, as revealed by the one-way ANOVA of the eating activity over the 45 min observation period (*F*_4,33_ = 30.09, *P* < 0.0001). The post hoc test demonstrated that both drugs at both dosages similarly affected this parameter (Figure 1(b)).

### 3.2. Effects of OEA and Rimonabant Administration on the Behavioral Satiety Sequence Pattern

**Figure 2:**
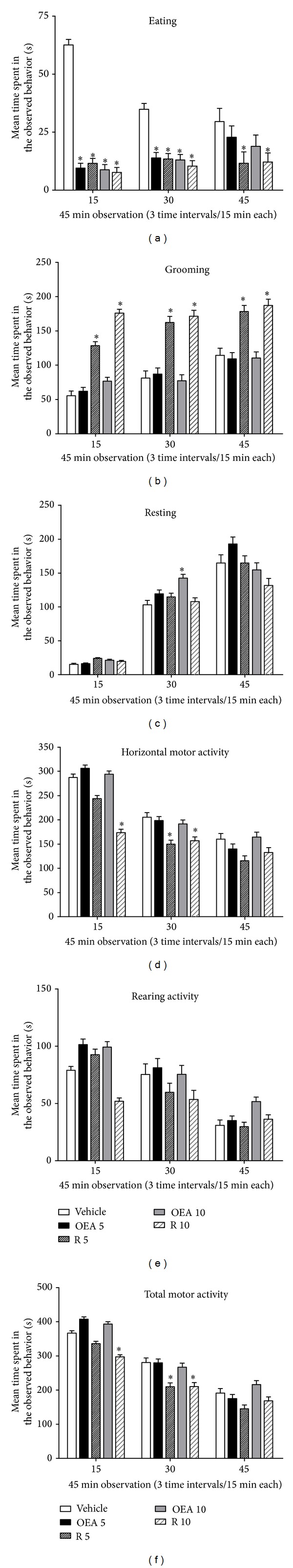
Panels illustrate the temporal development (duration of each behavior in sec) of eating (a), grooming (b), resting (c), horizontal motor activity (d), rearing activity (e), and total motor activity (f) in mice treated with vehicle, OEA 5 mg/kg i.p. (OEA 5), rimonabant 5 mg/kg i.p. (R 5), OEA 10 mg/kg i.p. (OEA 10), and rimonabant 10 mg/kg, i.p. (R 10). Represented on the* x*-axes are 3 time intervals of 15 min each for a total of 45 min of BSS analysis. Experiments were carried out during light-off period (08:00 PM-09:00 PM). *P < 0.05 versus vehicle group for each time interval (Tukey HSD test).

Two-way ANOVA analysis revealed a significant treatment effect on* eating activity *(*F*_4,33_ = 30.09, *P* < 0.0001) during the 45 min BSS testing. A significant treatment × time interaction (*F*_8,66_ = 615.46, *P* < 0.00001) was also found. Post hoc comparisons revealed a significant decrease of* eating activity *(Figure 2(a)) in OEA (5 and 10 mg/kg; both *P* < 0.001) and rimonabant (5 and 10 mg/kg; both *P* < 0.001) groups, as compared to controls, at 15 and 30 min time intervals. The reduction in* eating activity *was still evident for rimonabant at 45 min at both doses (5 and 10 mg/kg; both *P* < 0.005). The ANOVA analysis demonstrated a significant treatment effect on* grooming* (*F*_4,33_ = 65.02, *P* < 0.0001) as well as a significant treatment × time interaction (*F*_8,66_ = 2.13, *P* < 0.05). Post-hoc comparisons showed that rimonabant significantly increased grooming at both doses (5 and 10 mg/kg; *P* < 0.001) whatever the time interval considered, while OEA never affected the total time spent in grooming activity (Figure 2(b)). Concerning the time spent in* resting*, two-way ANOVA analysis evidenced a significant treatment effect (*F*_4,33_ = 5.73, *P* < 0.01) and a significant treatment × time interaction (*F*_8,66_ = 4.85, *P* < 0.0001). As further revealed by the post hoc analysis, with the exception of a significant and temporary (present only at the second, 30 min, time interval) increase in the time spent in* resting* in animals treated with OEA (10 mg/kg), no other significant changes were detected (Figure 2(c)). Concerning* locomotion* (horizontal motor activity, Figure 2(d)), the ANOVA analysis disclosed a significant treatment effect (*F*_4,33_ = 21.14, *P* < 0.0001) and a significant treatment × time interaction (*F*_8,66_ = 13.74, *P* < 0.0001). Post hoc comparisons showed that horizontal motor activity was decreased at 15 min interval in animals administered with rimonabant (10 mg/kg; *P* < 0.001), after 30 min interval in animals that received both doses of rimonabant (5 and 10 mg/kg; *P* < 0.01 and *P* < 0.005, resp.), while no significant changes were detected at the last time interval considered (45 min, Figure 2(d)). Moreover, two-way ANOVA analysis showed a significant treatment effect on* rearing *(*F*_4,33_ = 8.75, *P* < 0.0001) and a significant treatment × time interaction (*F*_8,66_ = 4.24, *P* < 0.001). Post hoc analysis evidenced that none of the drugs studied significantly affected the* rearing* activity. As showed (Figure 2(e)), although there was a tendency for rimonabant (10 mg/kg) to reduce rearing this reduction did not result significant. Finally, the ANOVA analysis on the total* motor activity* (Figure 2(f)) evidenced a significant main treatment effect (*F*_4,33_ = 22.64, *P* < 0.0001) and a significant treatment × time interaction (*F*_8,66_ = 4.64, *P* < 0.0001). Post hoc comparisons further showed that rimonabant induced a clear tendency to reduce total motor activity during the first time interval that became significant during the first time interval (15 min; 10 mg/kg; *P* < 0.01). Such decrease of total motor activity was evidenced also for the lower dose of rimonabant in the second (30 min) time interval (5 mg/kg; *P* < 0.01) (Figure 2(f)), while none of the drugs tested affected the whole motricity at the final time interval (45 min) analyzed (Figure 2(f)).

## 4. Discussion

The main finding of this study is that systemic administration of the same dosages of OEA or rimonabant produces similar anorexiant effects but different effects on other spontaneous behaviors related to feeding, such as grooming and resting. These similarities and differences may be of therapeutic relevance, since they might suggest that OEA is a possible pharmacological alternative to the CB1 antagonism/inverse agonism that might be devoid of the adverse effects observed with rimonabant.

Specifically, our results demonstrate that both drugs significantly reduced wet mash intake to a similar extent, with a clear dose-response fashion for OEA. This decrease was due to an overall similar decrease of eating activity recorded in the 45 min observation period. However, at the same time, the two drugs differed for the effects on other behaviors analyzed. In particular, grooming behavior was significantly affected by rimonabant treatment that caused a marked increase of the time spent in grooming throughout the 45 min period of observation (Figure 2(b)). Thus, although for the OEA-mediated effects the decrease of eating activity was less persistent than rimonabant, the general increase in grooming induced by rimonabant was identified all over the 45 min and no differences in the whole eating activity between the two drugs were detected (Figure 1(b)). Moreover, the grooming increase was paralleled by a decrease of locomotion (Figure 2(d)) and general activity (Figure 2(f)) particularly evident in mice treated with the highest dose of rimonabant. OEA treatment, besides decreasing eating activity within the first 30 min of observation, caused also a delayed increase (evident at 30 min) of the time spent resting in mice treated with the highest dose (Figure 2(c)). However, such increase was not accompanied by any decrease of horizontal (locomotion), vertical (rearing), and total motor activity whatever the dose tested.

The effects of rimonabant on grooming behavior in rodents have been described in the literature with similar observations made also for the preclinical effects of other CB1 receptor antagonists/inverse agonists and even newer neutral CB1 receptor antagonists [50, 53]. In particular several studies described a syndrome of compulsive scratching and grooming in rodents as well as severe itching and scratching in humans [14, 67, 68]. Initially such effects were considered to be responsible for the anorexiant actions of the drugs, due to a sort of response competition between eating and grooming/scratching behaviors. However, it has been demonstrated that the two actions are independent and probably mediated by the interactions of rimonabant with different (central versus peripheral) receptors and the involvement also of other mechanisms such as the activation of opioid receptors [14].

In our study we did not focus on scratching behaviour but did record an intense compulsive grooming in mice treated with rimonabant that gave us the idea of a competitive behavior able to interfere with eating activity. However, testing this hypothesis was beyond the aim of our experiments.

Compulsive grooming in mice has an important ethological relevance. It is particularly sensitive to stress and might indicate an altered emotional reactivity of the animal [69]. The stimulation of grooming behavior caused by rimonabant treatment is compatible with its anxiogenic effects observed in several animal models [54]. By contrast, this effect was completely absent in animals treated with OEA. We previously demonstrated that OEA decreases food intake in free-feeding rodents by causing a dose-dependent delay in eating onset, which is not accompanied by changes in meal size or postmeal interval [32]. This delay cannot be attributed to motoric inhibition, because it occurs at doses of OEA (5–10 mg/kg) that have no effect on either locomotor activity in the open field test or operant responding for food [31]. Furthermore, the OEA-induced delay in feeding is unlikely to be caused by anxiety or malaise since OEA does not alter rodent performance in the elevated plus-maze test or produce conditioned taste aversion for saccharin [31].

OEA actions seem to be associated with the activation of PPAR-*α* receptors, as its anorexiant effects, as well as those reported on the meal pattern, have not been observed in PPAR-*α* null mice [29]. Other possible receptors suggested to be involved in OEA's actions are the capsaicin receptor TRPV1 and the G protein coupled receptor GPR119 [70–72]. However, the observation that genetic deletion of TRPV1 or GPR119 in mice does not prevent OEA anorexiant actions [41, 73], as observed in mice lacking PPAR-*α* receptors [29], strongly supports the hypothesis that PPAR-*α* receptors mediate OEA effects on feeding behaviour.

Peripheral rather than central PPAR-*α* receptors seem to be particularly involved. This hypothesis is supported by the observation that OEA anorexiant action is completely absent when peripheral sensory fibers are removed by capsaicin treatment, by surgical dissection or when the drug is injected into the brain ventricles [31]. However, OEA can still inhibit feeding when locally infused in the rat lateral hypothalamus [74] and OEA and PPAR-*α* can be found in the brain, where they play a role in the modulation of several functions including cognition [75] drug and food reward [46, 76] neuroprotection [77] and sleep-waking cycle [74]. Nevertheless, the finding that systemic administration of OEA is not followed by increased levels of the drug in the brain [78] suggests that peripheral and central compartments of OEA distribution are kept separated. This is presumably attributable to the high expression of its degrading enzyme, fatty acid amide hydrolase, in the blood-brain barrier [79].

The same enzyme is responsible also for the degradation of AEA, which in many of the cited functions of OEA may be seen as a functional antagonist. This is particularly evident for the modulation of food intake and lipid metabolism, for which AEA and OEA play two opposite roles: AEA stimulates appetite and promotes lipogenesis and body weight gain, while OEA inhibits appetite, stimulates lipolysis, and decreases body weight gain.

## 5. Conclusions

Based on these considerations, the results of the present study corroborate the idea that OEA [24, 25, 80] might represent a novel anorexiant agent sharing with rimonabant similar powerful effects on food intake but not the same impact on feeding behavior. In contrast to rimonabant, OEA does not block CB1 receptors and is not accountable for the wide spectrum of unwanted effects typically observed with CB1 antagonists/reverse agonists, whereas, by acting as a functional antagonist, it might counteract the actions of ECs on appetite stimulation and on the control of energy balance. We have recently showed that OEA administration can induce satiety by indirectly stimulating oxytocin neurosecretion from the hypothalamus [30, 81, 82], thus further supporting the notion that OEA or OEA-related compounds might represent a novel and safer antiobesity agent [83, 84] targeting systems other than those controlled by CB1 activation/blockade.
